# Breast metastasis from pelvic high-grade serous adenocarcinoma: a report of two cases

**DOI:** 10.1186/s40792-020-01090-7

**Published:** 2020-12-09

**Authors:** Yurina Harada, Makoto Kubo, Masaya Kai, Mai Yamada, Karen Zaguirre, Tatsuhiro Ohgami, Hideaki Yahata, Yoshihiro Ohishi, Hidetaka Yamamoto, Yoshinao Oda, Masafumi Nakamura

**Affiliations:** 1 Department of Surgery and Oncology, Graduate School of Medical Sciences, Kyushu University, 3-1-1 Maidashi, Higashi-ku, Fukuoka, 812-8582 Japan; 2 Department of Obstetrics and Gynecology, Graduate School of Medical Sciences, Kyushu University, 3-1-1 Maidashi, Higashi-ku, Fukuoka, 812-8582 Japan; 3 Department of Diagnostic Pathology, Aso Iizuka Hospital, 3-83 Yoshiomachi, Iizuka, Fukuoka 820-8501 Japan; 4 Department of Anatomic Pathology, Graduate School of Medical Sciences, Kyushu University, 3-1-1 Maidashi, Higashi-ku, Fukuoka, 812-8582 Japan

**Keywords:** Peritoneal cancer, Breast metastasis, Core needle biopsy, Hereditary breast and ovarian cancer syndrome

## Abstract

**Background:**

Metastatic tumors to the breast reportedly account for 0.5% to 2.0% of all malignant breast diseases. Such metastatic tumors must be differentiated from primary breast cancer. Additionally, few reports have described metastases of gynecological cancers to the breast. We herein report two cases of metastasis of pelvic high-grade serous adenocarcinoma to the breast.

**Case presentation:**

The first patient was a 57-year-old woman with a transverse colon obstruction. Colostomy was performed, but the cause of the obstruction was unknown. We found scattered white nodules disseminated throughout the abdominal cavity and intestinal surface. Follow-up contrast-enhanced computed tomography (CT) showed an enhanced nodule outside the right mammary gland. Core needle biopsy (CNB) of the right breast mass was conducted, and immunohistochemical staining of the mass suggested a high-grade serous carcinoma of female genital tract origin. We diagnosed the patient’s condition as breast and lymph node metastasis of a high-grade serous carcinoma of the female genital tract. After chemotherapy for stage IVB peritoneal cancer, tumor reduction surgery was performed. The second patient was a 71-year-old woman with a medical history of low anterior resection for rectal cancer at age 49, partial right thyroidectomy for follicular thyroid cancer at age 53, and left lower lung metastasis at age 57. Periodic follow-up CT showed peritoneal dissemination, cancerous peritonitis, and pericardial effusion, and the patient was considered to have a cancer of unknown primary origin. Contrast-enhanced CT showed an enhanced nodule in the left mammary gland with many enhanced nodules and peritoneal thickening in the abdominal cavity. CNB of the left breast mass was conducted, and immunohistochemical staining of the mass suggested a high-grade serous carcinoma of female genital tract origin. After chemotherapy for stage IVB peritoneal cancer, tumor reduction surgery was performed.

**Conclusions:**

We experienced two rare cases of intramammary metastasis of high-grade serous carcinoma of female genital tract origin. CNB was useful for confirming the histological diagnosis of these cancers that had originated from other organs. A correct diagnosis of such breast tumors is important to ensure quick and appropriate treatment.

## Background

Metastatic tumors to the breast reportedly account for approximately 0.5% to 2.0% of all malignant breast diseases [1]. Overall, breast metastases from other organs are rare, especially breast metastases from gynecological malignant diseases. To our knowledge, only seven such cases were reported in Japan from 1997 to 2018 (Table 1) [2–8]. The distinction between primary breast cancer and metastatic tumors to the breast is extremely important in determining the treatment strategy. A core needle biopsy (CNB) is one of the most useful diagnostic examinations to determine whether a breast tumor is primary or metastatic. We experienced two cases of breast metastasis from high-grade serous carcinoma of female genital tract origin. In both cases, the diagnosis was made by CNB of an intramammary mass.

**Table 1:** Reported cases of metastatic ovarian cancer to the breast (in Japan)

Case	First author	Date	Age (years)	Type of ovarian tumor	Survival	Therapy
1.	Tachibana [2]	1998	52	Moderately differentiated adenocarcinoma	3 months	Unknown
2.	Maruyama [3]	2007	43	Clear cell adenocarcinoma	Unknown	Chemotherapy
3.	Yoshii [4]	2009	71	Serous poorly differentiated adenocarcinoma	Unknown	Chemotherapy + surgery
4.	Suehiro [5]	2012	46	Clear cell carcinoma	1 year	Chemotherapy + surgery
5.	Takao [6]	2016	60	High-grade serous adenocarcinoma	> 1 year	Chemotherapy + surgery
6.	Ikari [7]	2017	51	Serous papillary adenocarcinoma	> 10 months	Chemotherapy + surgery
7.	Kimura [8]	2018	68	Clear cell adenocarcinoma	15 months	Chemotherapy + surgery
8.	Current case 1	2018	57	High-grade serous adenocarcinoma	> 9 months	Chemotherapy + surgery
9.	Current case 2	2018	71	High-grade serous adenocarcinoma	> 10 months	Chemotherapy + surgery

## Case presentation

### Case 1

The first patient was a 57-year-old woman with no relevant medical history. She was evaluated for vomiting, abdominal pain, and weight loss caused by a transverse colon obstruction. Colostomy was performed at another hospital, but the cause of the obstruction was unknown. Cytology of the ascites indicated the presence of poorly differentiated adenocarcinoma cells, and scattered white nodules were found to be disseminated throughout the abdominal cavity and intestinal surface; however, there was no evidence of a tumor in the uterus or ovaries. The patient was referred to our hospital for detailed examination and treatment. After the colostomy, she had lost 10 kg of body weight within 1 month.

**Fig. 1:**
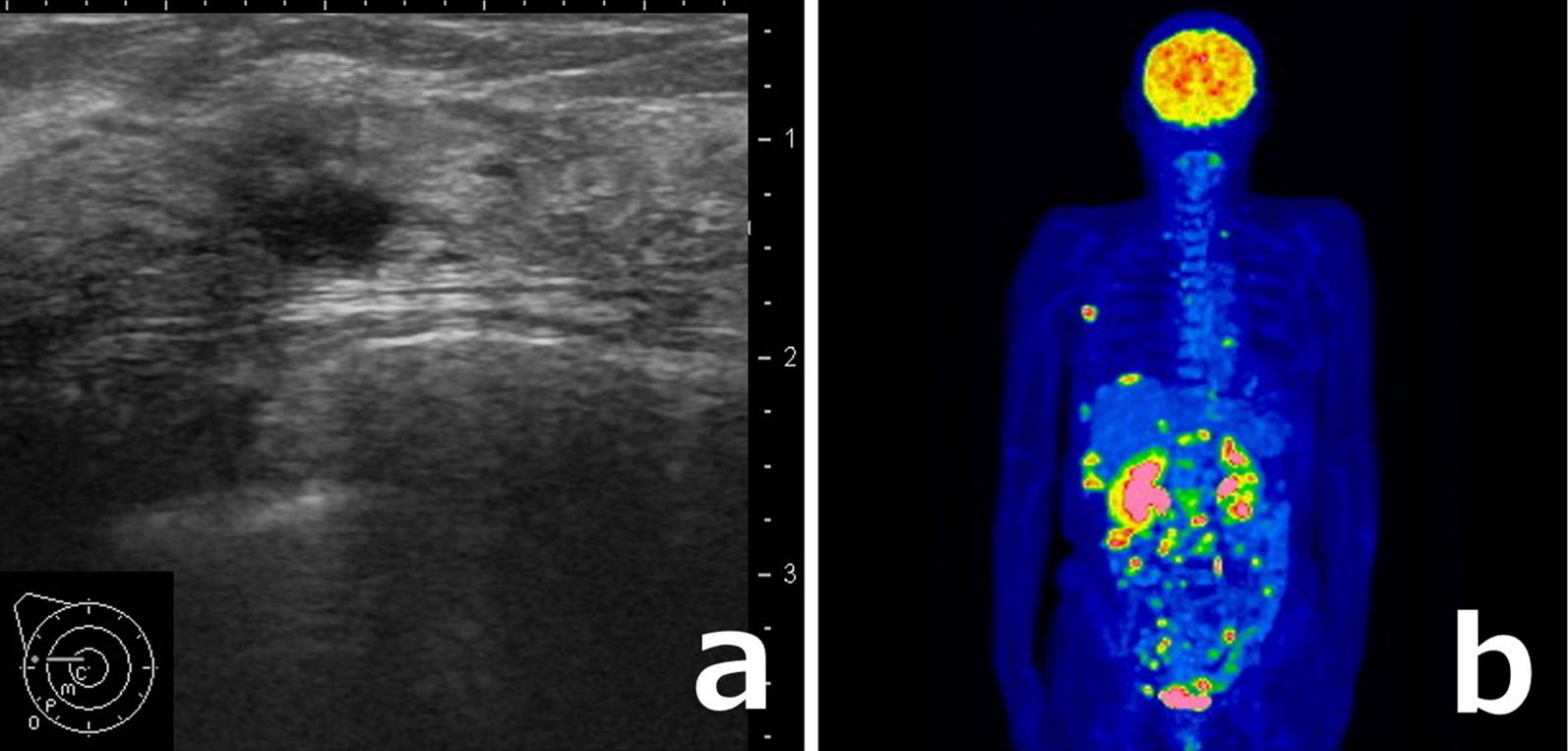
Case 1: imaging findings. **a** Ultrasound revealed a low-echo area of approximately 5 mm in the upper-outer quadrant of the right breast. **b** Positron emission tomography–computed tomography showed numerous foci of dissemination and lymphadenopathy in the abdominal cavity, including the intestine directly under the artificial anus, the right lumbar subcutaneous region, and the right uterine appendages

Blood tests revealed elevated CA125 and CA15/3 levels of 427 U/ml and 54.7 U/ml, respectively. Other tumor markers CEA and CA19-19 were within the normal ranges at 3.5 ng/ml and 8 U/ml, respectively. Mammography showed no significant findings. Ultrasound revealed multiple hypoechoic lesions of approximately 5 mm in the upper-outer quadrant of the right breast (Fig. 1a). Contrast-enhanced computed tomography (CT) showed an enhanced nodule of about 5 mm outside the right mammary gland. Large and small tumors were found in the abdominal pelvis, with moderate ascites retention and lymphadenopathy around the right groin and abdominal aorta. Contrast-enhanced magnetic resonance imaging revealed many nodules in the abdominal wall, which suggested metastasis and disseminated lesions. No obvious swelling was present in the uterine appendages. The right inguinal lymph node and the obturator external iliac lymph node were swollen. Positron emission tomography–CT showed numerous foci of dissemination and lymphadenopathy in the abdominal cavity, such as the right subdiaphragm, colostomy intestine, right lumbar subcutaneous region, right inguinal region, and right uterine appendages. Abnormal accumulation was also found in the right supraclavicular fossa, right axilla, mediastinal lymph node, right chest wall, and pericardium (Fig. 1b). Many lesions that appeared to be metastatic foci, including a breast mass, were observed; however, no clear primary lesions were identified on the image. Thus, we considered the patient to have a cancer of unknown primary origin and performed further examinations.

**Fig. 2:**
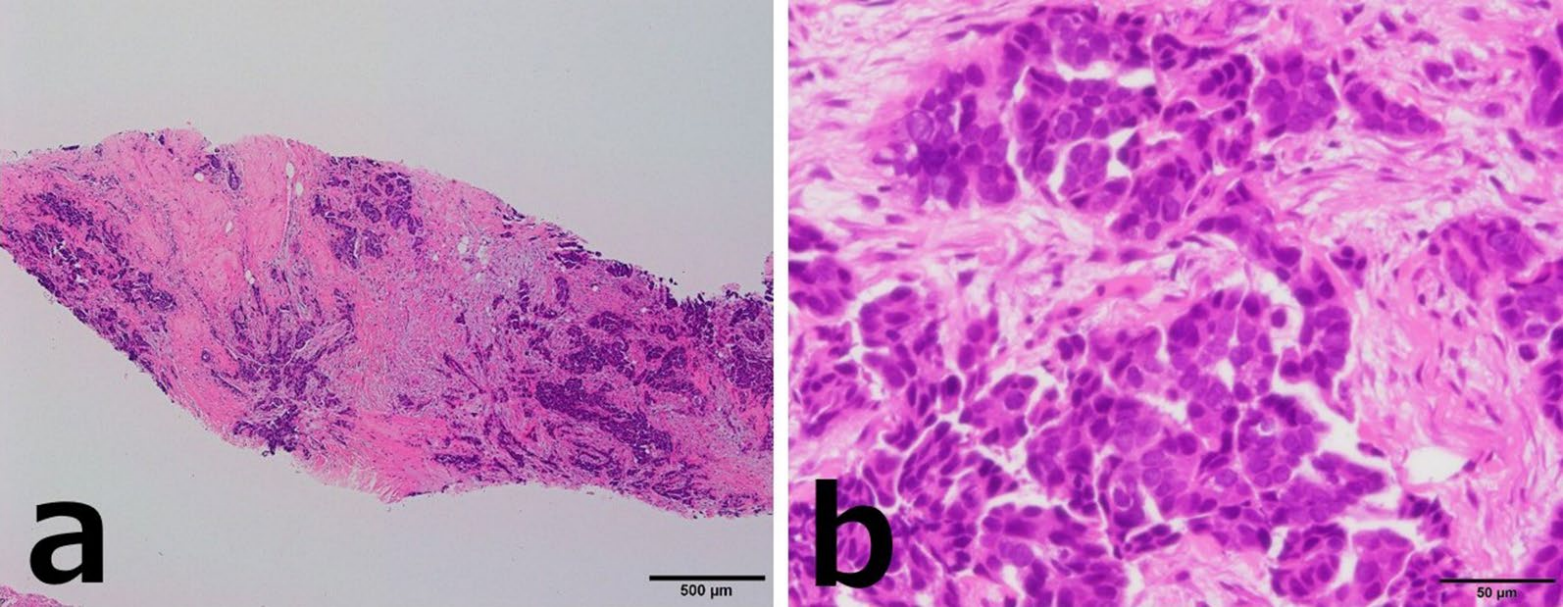
Case 1: histopathological findings of the breast tumor (hematoxylin/eosin staining). **a** ×40. **b** ×400. Carcinoma cells with eosinophilic cytoplasm were arranged in irregular nests invading the stroma

**Fig. 3:**
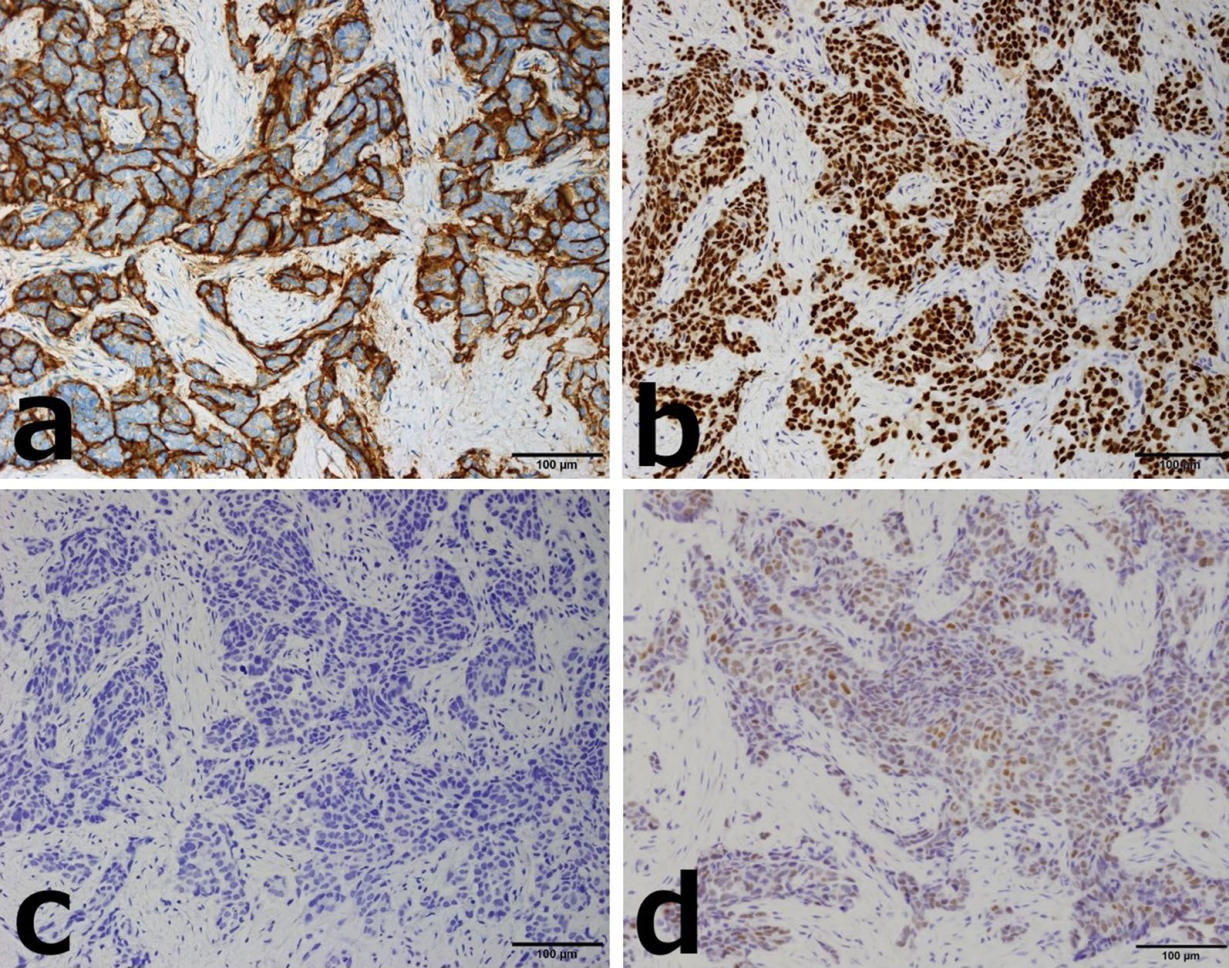
Case 1: breast tumor histopathology (immunostaining). **a** CA125. **b** WT-1. **c** GCDFP15. **d** PAX-8. Immunostaining revealed diffuse positivity for CA125, WT-1, and PAX-8, and negativity for GCDFP15, suggesting a high-grade serous carcinoma derived from the uterus, ovary, fallopian tube, or peritoneum

CNB of the right breast mass revealed cancer cells with eosinophilic cytoplasm arranged in irregular nests invading the stroma (Fig. 2a, b). Immunostaining revealed diffuse positivity for p53, CA125, WT1, and PAX-8 and negativity for GCDFP15, suggesting a high-grade serous carcinoma derived from the uterus, ovary, fallopian tube, or peritoneum (Fig. 3a–d).

**Fig. 4:**
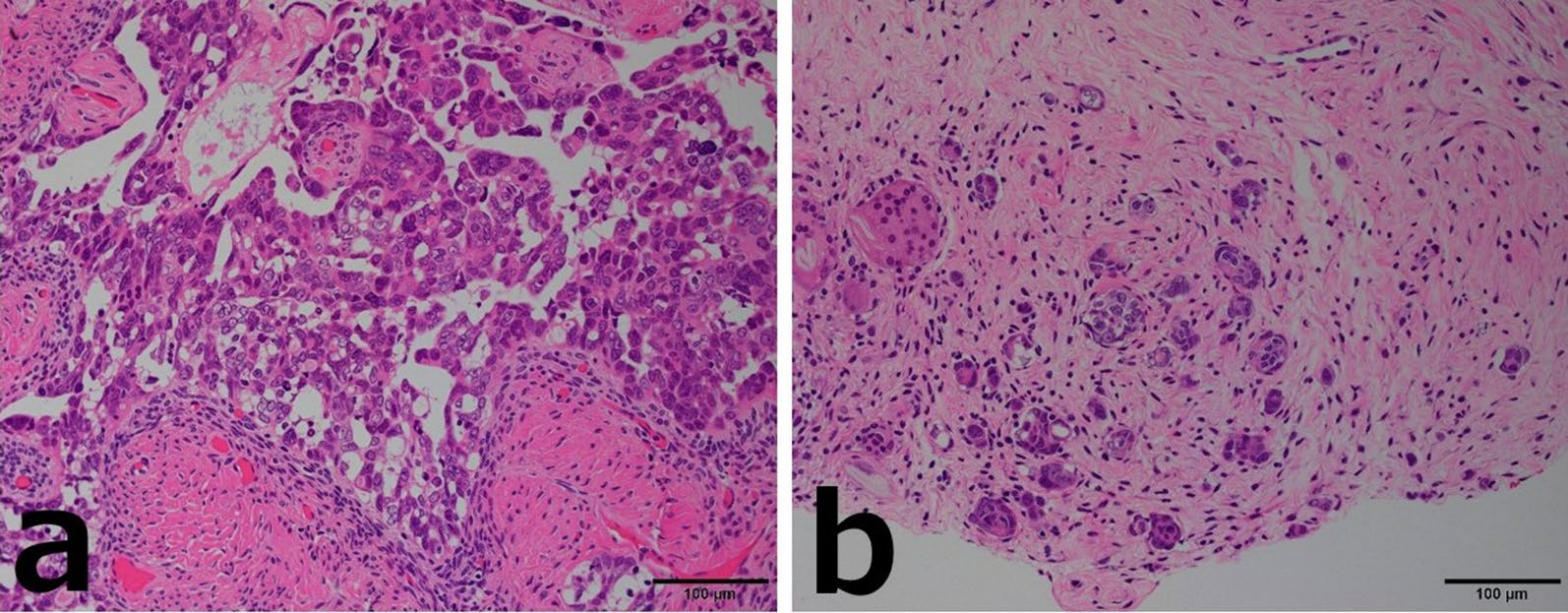
Case 1: microscopic findings of the resected specimen (hematoxylin/eosin staining). **a** Ovarian histopathology. **b** Peritoneal histopathology. Both are similar to the histopathologic findings of breast tumors, with carcinoma cells arranged in irregular glands, micropapillary structures, and a small nested and slit-like pattern

Based on the pathological findings of the mammary lesions, we diagnosed the patient’s condition as breast and lymph node metastases of a high-grade serous carcinoma of female genital tract origin. Considering all findings, this case was diagnosed as peritoneal cancer based on the Gynecologic Oncology Group (GOG) criteria (Table 2) [9]. After six courses of ddTC chemotherapy [dose-dense Taxol (paclitaxel) + carboplatin] for stage IVB peritoneal cancer, tumor reduction surgery was performed. The pathological findings of the resected ovary and peritoneum were similar to those of the breast tumors, with carcinoma cells arranged in irregular glands and a small nested and slit-like pattern (Fig. 4a, b). The bilateral fallopian tubal tissues are free of carcinoma cells. After surgery, six courses of ddTC chemotherapy were performed as adjuvant therapy. Four months after the end of treatment, the patient developed peritoneal dissemination and multiple lymph node metastases.

**Table 2:** Diagnostic criteria for primary peritoneal cancer (GOG)

1. Bilateral ovaries are normal size or swollen because of benign changes
2. Extraovarian lesions are larger than those on the surface of the ovary
3. Microscopically, the ovarian lesions meet one of the following:
No lesion in ovary
Lesion was confined to the superficial epithelium of the ovary without invasion of the stroma
Lesions within the ovarian surface epithelium and stroma, but within 5 × 5 mm
The lesion within the parenchyma of the ovary is within 5 × 5 mm regardless of the presence or absence of lesions on the surface of the ovary
4. The histological and cytological features of the tumor are similar or identical to serous adenocarcinoma of the ovary

*GOG* Gynecologic Oncology Group

### Case 2

The second patient was a 71-year-old woman with a medical history of low anterior resection for primary rectal cancer at age 49, partial right thyroidectomy for primary follicular thyroid cancer at age 53, and left lower lung metastasis of rectal cancer at age 57. A left lower lobectomy was performed for the metastatic lung tumor. Her father had colorectal cancer.

The chief complaint at the initial visit was abdominal distension. Periodic follow-up CT showed peritoneal dissemination, cancerous peritonitis, and pericardial effusion. Recurrence of rectal cancer was suspected, but the findings were atypical. Therefore, the patient was further evaluated for cancer of unknown primary origin.

**Fig. 5:**
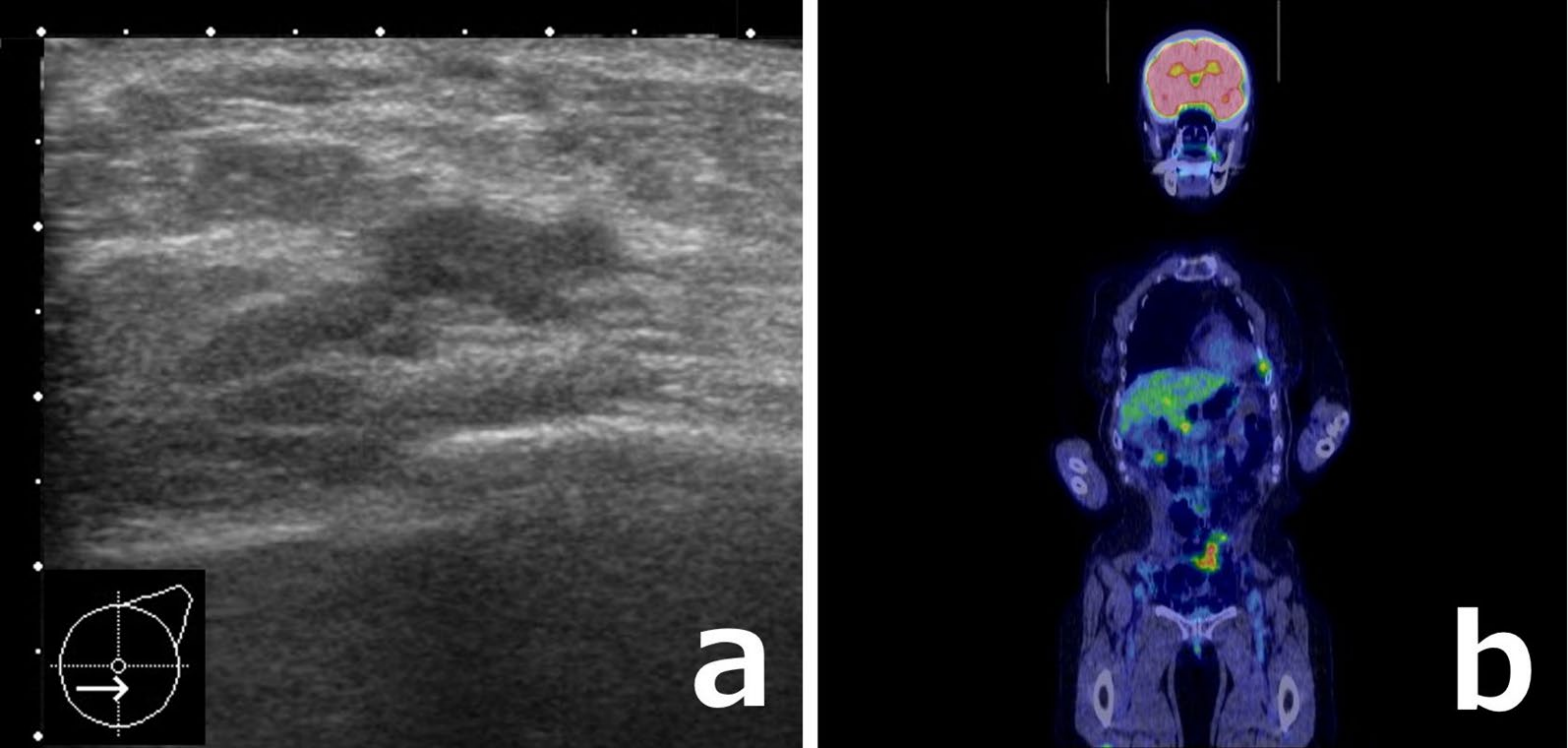
Case 2: imaging findings. **a** Ultrasound revealed a low-echo area of 18 × 6 × 13 mm with an unclear boundary in the lower-inner quadrant of the left breast. **b** Positron emission tomography–computed tomography showed abnormal accumulation in the rectal excision, peritoneal thickening in the pelvis, and other nodules in the abdominal cavity

Blood tests revealed elevated CA125 and CA15-3 levels of 966.4 U/ml and 106.4 U/ml, respectively. CEA and CA19-9 levels were within the normal ranges at 1.0 ng/ml and 24.8 U/ml, respectively. Mammography showed no significant findings. Ultrasound revealed a hypoechoic lesion of 18 × 6 × 13 mm with an unclear boundary in the lower-inner quadrant of the left breast (Fig. 5a). Contrast-enhanced CT showed an enhanced nodule of approximately 15 mm in the left lower-inner mammary gland, many enhanced nodules, mesenteric adipose opacity, and peritoneal thickening in the abdominal cavity. A small amount of ascites and pericardial effusion were found in the pelvis and liver margin. Positron emission tomography–CT showed abnormal accumulation in the rectal excision, peritoneal thickening in the pelvis, and other nodules in the abdominal cavity (Fig. 5b). Many lesions that appeared to be metastatic foci, including a breast mass, were observed; however, no clear primary lesions were identified on imaging. Therefore, we considered the patient to have a cancer of unknown primary origin and performed further examinations.

**Fig. 6:**
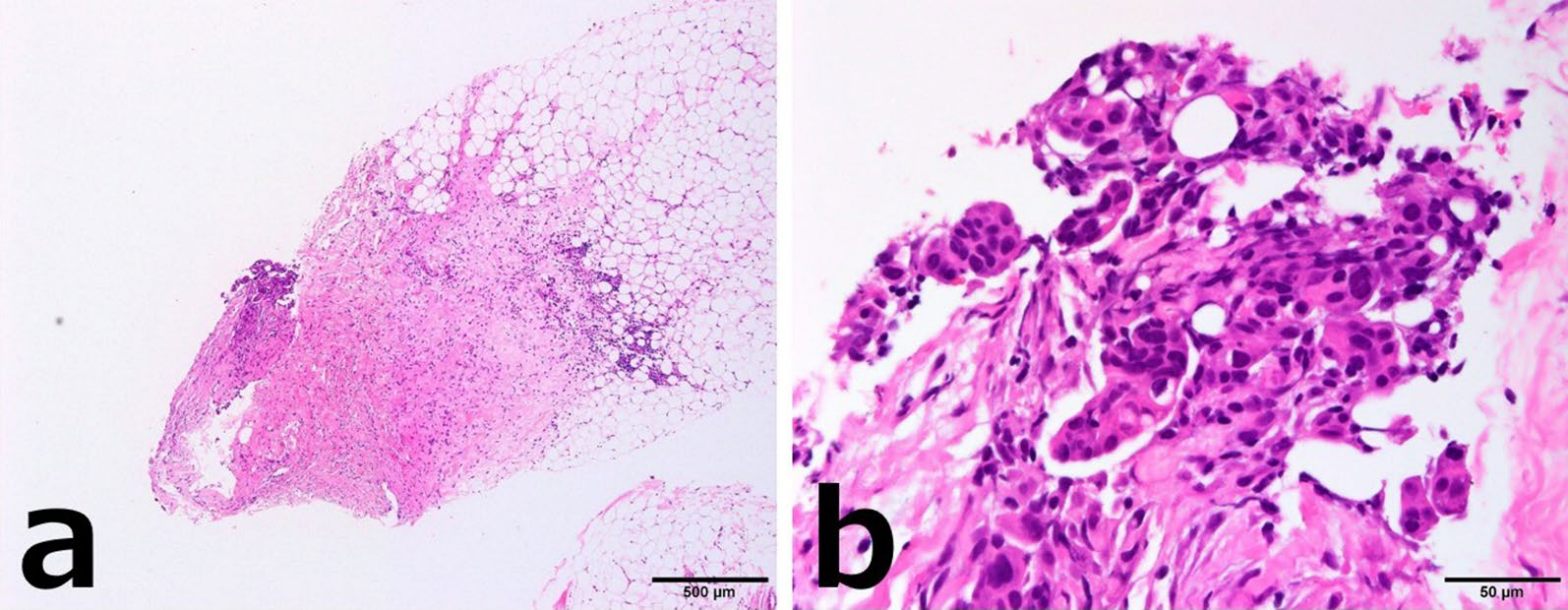
Case 2: breast tumor histopathology (hematoxylin/eosin staining). **a** ×40. **b** ×400. Vesicles of poorly differentiated cancer cells were present

**Fig. 7:**
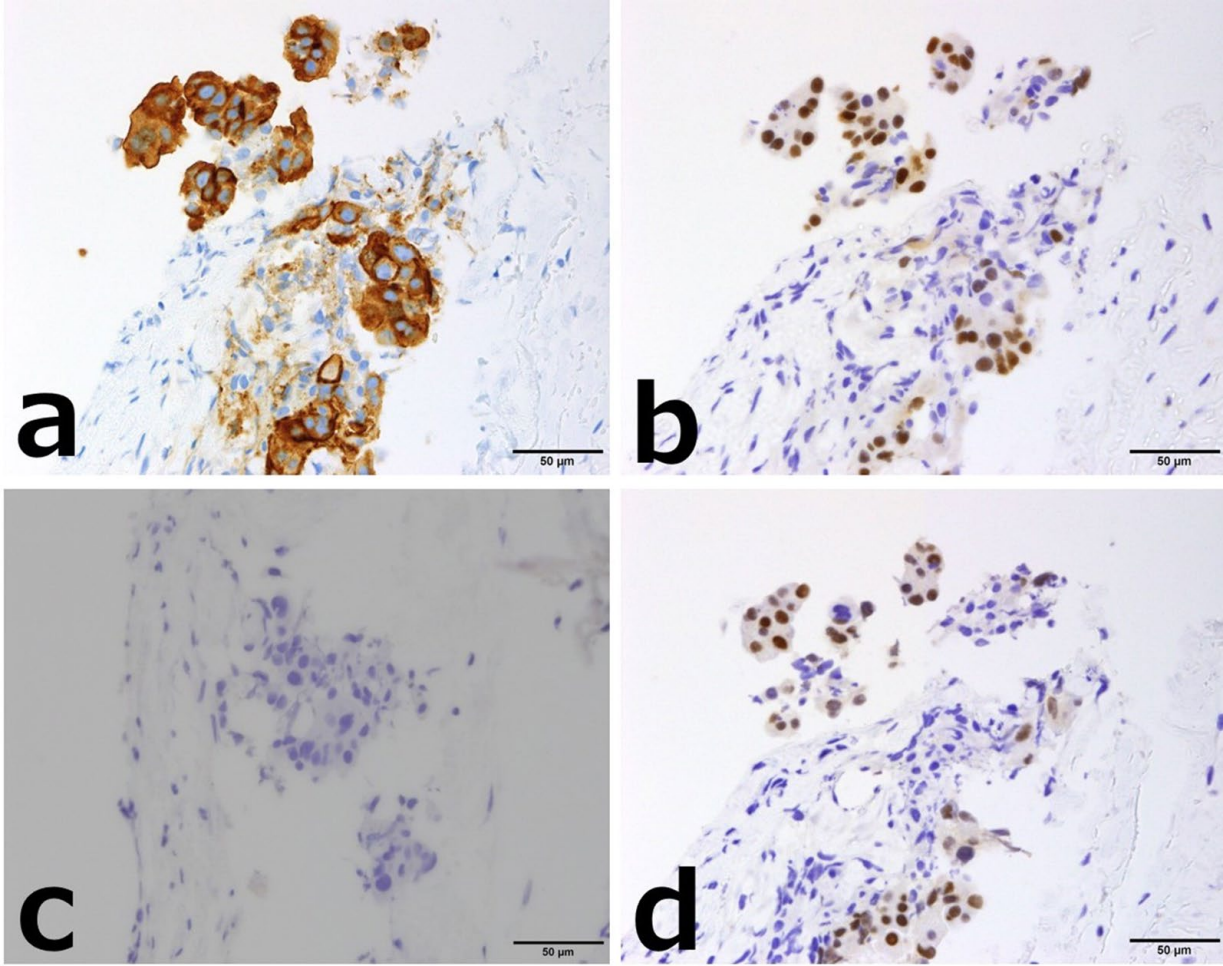
Case 2: breast tumor histopathology (immunostaining). **a** CA125. **b** WT-1. **c** GCDFP15. **d** PAX-8. Immunostaining showed positivity for CA125, WT-1, and PAX-8, and negativity for GCDFP15. Notably, PAX-8 is highly specific for Müllerian tube-derived cancer cells and was positive in this case

CNB of the left breast mass revealed vesicles of poorly differentiated cancer cells (Fig. 6a, b). Immunostaining showed positivity for AE1/AE3, PAX-8, CA125, and WT1, and negativity for TTF1, CDX2, and GCDFP-15 (Fig. 7a–d).

**Fig. 8:**
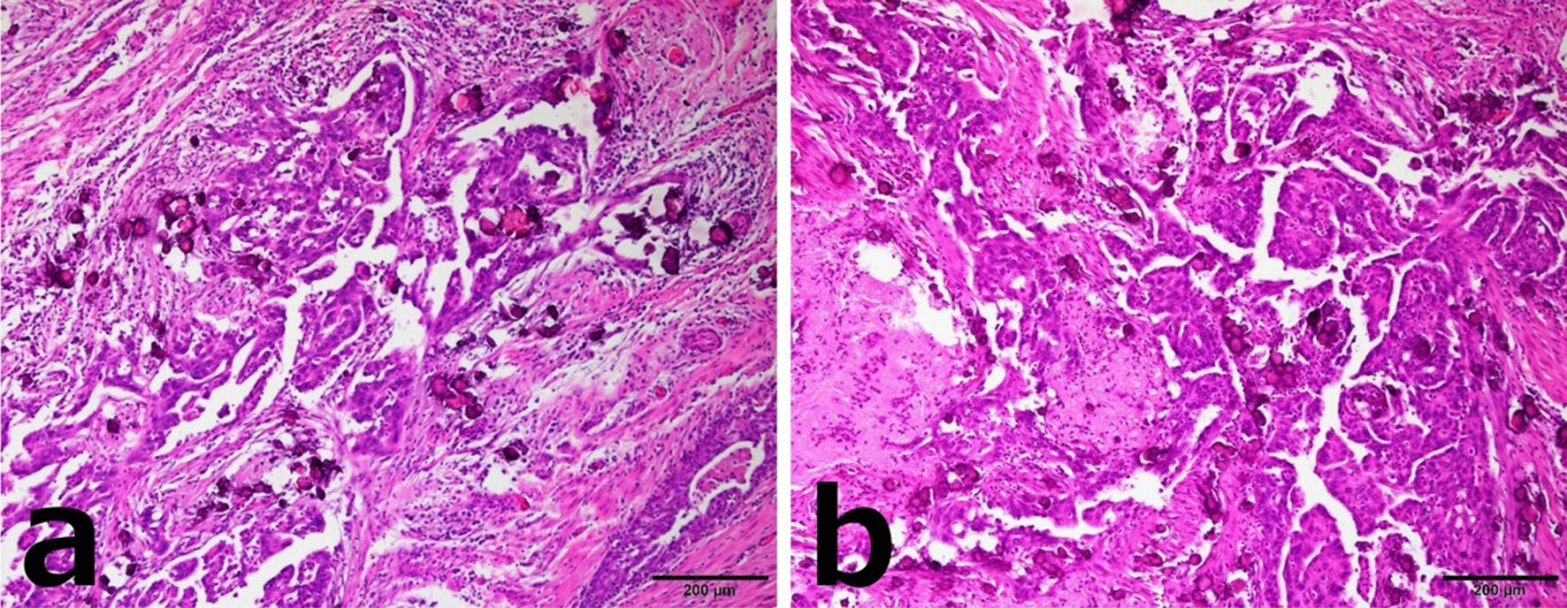
Case 2: microscopic findings of the resected specimen (hematoxylin/eosin staining). **a** Ovarian histopathology. **b** Omental histopathology. Examination revealed the proliferation of adenocarcinoma cells with a basophilic cytoplasm and strong atypical nuclei

Based on the pathological findings of the mammary lesions, we diagnosed the patient’s condition as breast and lymph node metastases of a high-grade serous carcinoma of female genital tract origin. Considering all findings, this case was diagnosed as peritoneal cancer based on the GOG criteria (Table 2) [9]. After four courses of ddTC chemotherapy for stage IVB peritoneal cancer, tumor reduction surgery was performed (Fig. 8a, b). After surgery, six courses of docetaxel + carboplatin chemotherapy were performed as adjuvant therapy. Fourteen months after the end of treatment, the patient developed brain metastasis.

## Conclusions

Primary peritoneal cancer is a multicentric tumor that develops from mesothelial cells covering the omentum, diaphragm, mesentery, and ovarian superficial epithelial cells that are continuous with these cells. It shows the same pathological condition as superficial epithelial/interstitial malignant tumor of the ovary and is mostly serous adenocarcinoma. The GOG diagnostic criteria are most widely used for the diagnosis of peritoneal cancer (Table 2) [9].

Gynecological cancers are malignant tumors that develop in the female genital organs, specifically the vulva, vagina, uterus, fallopian tubes, and ovaries. The most common gynecological cancer types are ovarian, cervical, and endometrial cancer. Serous adenocarcinoma often occurs in the ovary, uterine body, fallopian tube, and peritoneum of gynecological tissues and most frequently affects the ovary (36%) and endometrium (5.9%); cervical involvement is very rare [10]. Fallopian tube and peritoneal cancers themselves are rare, accounting for approximately 1% of gynecological cancers [11]. Therefore, if serous adenocarcinoma of the pelvis is observed, we first consider the ovary or uterus as the primary focus. But in our two cases, no significant findings were observed in the ovary or uterus on imaging, and both peritoneal dissemination and cancerous peritonitis were noted. That is why we considered the possibility of peritoneal cancer.

The principle of the treatment for serous adenocarcinoma of the pelvis is multidisciplinary therapy involving a combination of tumor reduction surgery and chemotherapy. Treatment is performed as for so-called Müllerian adenocarcinoma together with epithelial ovarian cancer and fallopian tube cancer [12]. As chemotherapy for pelvic serous adenocarcinoma, TC therapy (paclitaxel at 175 mg/m^2^ on day 1, carboplatin at AUC 5 on day 1, over a 21-day cycle) is the first-choice treatment according to established guidelines such as those of the National Comprehensive Cancer Network [13] and the American Society of Clinical Oncology [14]. No treatment that greatly exceeds TC therapy has yet been found; however, ddTC therapy, in which paclitaxel is administered in divided doses, increases the total relative dose intensity and has been widely used. In addition, prolongation of progression-free survival was observed by combining TC therapy with the vascular endothelial growth factor antibody bevacizumab, which is a molecular-targeted drug. Furthermore, it has been shown to be effective in controlling the ascites associated with cancerous peritonitis [15].

High-grade serous adenocarcinoma grows very efficiently in the abdominal cavity. Retroperitoneal lymph node metastases are present in more than 50% of cases, which have a poor prognosis [16]. Also, approximately 50% have microscopic metastases to the uterus, and about 30% have macroscopic metastases to the uterus [17], but it rarely spreads outside the abdominal cavity. Most patients who had peritoneovenous shunts that infused cancer cells into the venous system through the jugular vein did not develop disseminated hematogenous metastases after up to 2 years of continuous shunting [18].

Based on the above, breast metastasis of serous adenocarcinoma from the pelvis is extremely rare, and in fact, has rarely been reported. According to a report by Hajdu and Urban [19], a metastatic tumor to the breast is characterized by histologic features similar to those of the primary lesion; additionally, the existing mammary gland tissue is not destroyed, and findings of intraductal carcinoma and noninvasive lobular carcinoma are present. Another report stated that malignant cells are distributed around the breast ducts and lobules [20]. In clinical practice, it is difficult to distinguish primary breast cancer from metastatic breast cancer using hematoxylin–eosin (HE) staining. Breast tumors can be highly diagnosed as primary breast cancer if they have a component of ductal carcinoma in situ, but if they have no component, they cannot be diagnosed either. This is the reason why a diagnosis of primary or metastatic lesions is usually performed using immunostaining. In both of our cases, microscopic examination of the breast tumor biopsy specimens revealed no intraductal carcinoma, and proliferations of carcinoma cells were arranged in irregular nests invading the stroma around the lobules.

However, approximately 81% of ovarian cancers in patients with hereditary breast and ovarian cancer (HBOC) caused by *BRCA1*/*BRCA2* gene variants are reportedly serous adenocarcinoma [21]. Therefore, when tumors in the pelvis and breast are recognized, HBOC should be taken in account even in our two patients. We considered the possibility of multiple cancers, including breast cancer, because the CA15-3 level was elevated, and some characteristic image findings were present in the pelvis and breast. As a result, the pathological diagnosis of the breast tumor was serous adenocarcinoma in both of our patients.

*BRCA1*/*BRCA2* gene testing was not performed in either patient thus far, but, if pathogenic variants are detected by genetic testing, the patient should be provided with appropriate genetic counseling for HBOC. *BRCA1*/*BRCA2* gene testing is particularly relevant for indications of PARP inhibitors. Recommendations for *BRCA1*/*BRCA2* gene testing vary with each guideline. The National Comprehensive Cancer Network (NCCN) suggests that all ovarian cancer patients, regardless of family history, should be considered for *BRCA1*/*BRCA2* gene testing [22], whereas the European Society for Medical Oncology (ESMO) suggests that they should be considered according to a family history of breast and ovarian cancer [23]. In Japan, the PARP inhibitor olaparib was approved as a “maintenance therapy for recurrent ovarian cancer with sensitivity to platinum-based antineoplastic agents” in 2018. In randomized phase 3 trials of PARP inhibitors, the NOVA trial [24] and ARIEL3 [25], progression-free survival (PFS) was significantly prolonged in platinum-sensitive recurrent ovarian cancer with and without *BRCA* mutations. Later, olaparib was also approved as “maintenance therapy after initial treatment for *BRCA* mutation-positive ovarian cancer”. However, when used for the treatment of recurrent ovarian cancer as well as for peritoneal cancer, the duration of response to platinum-based antineoplastic agents is used as an indicator of whether PARP inhibitors are effective. The gynecologists in our hospital evaluated the durations of clinical benefit that carboplatin provided to both of our patients (14 and 23 months, respectively) were not so long compared with the median PFS resulting from ddTC therapy of 28.2 months in the JGOG 3016 trial [26]. The PFS of case 2 is close to that of the ddTC group in the ICON8 trial (24.9 months) [27], but it is still a short period of time. Therefore, it could be possible that both of our two cases were platinum-resistant, and they were unlikely to be patients with HBOC regarding their family history. In addition, *BRCA1/BRCA2* gene test was not covered by health insurance as a companion diagnostic test for PARP inhibitor administration for ovarian cancer in Japan, so we did not perform it in our two cases. Conversely, it is not performed for most patients with platinum-sensitive ovarian cancer as well.

In summary, we experienced two rare cases of metastatic breast tumors diagnosed as high-grade serous carcinoma of female peritoneal cancer origin. In both cases, CNB of the breast tumors was useful for achieving a precise diagnosis. The diagnoses were made by pathologists who were familiar with both female genital tract cancer and breast cancer. Confirming whether a breast tumor is primary or metastatic is important because the treatment and prognosis totally differ between the two conditions.

## Data Availability

Data sharing is not applicable to this article because no datasets were generated or analyzed during the current study.

## Abbreviations

CNB: Core needle biopsy
CT: Computed tomography
ddTC: Dose-dense paclitaxel + carboplatin
HBOC: Hereditary breast and ovarian cancer syndrome
PFS: Progression-free survival
GOG: Gynecologic Oncology Group

## References

[1] Chaingnaud B, Hall TJ, Powers C, Subramony C, Scott-Conner CE (1994). Diagnosis and natural history of extramammary tumors metastatic to the breast. J Am Coll Surg.

[2] Tachibana A, Fukuma E, Ui Y, Yamakawa T, Mizuguchi K (1998). A case of ovarian cancer with breast metastasis. J Jpn Surg Assoc.

[3] Maruyama S, Masuda H, Fujii T, Kunisue H, Kanaya Y, Yokoyama N (2007). A case of breast metastasis from ovarian cancer. J Jpn Surg Assoc.

[4] Yui T, Ishitani K, Konno J, Fukagawa F, Ueda E, Nomura H (2009). A case of ovarian serous adenocarcinoma with breast metastasis. Tokyo J Obstet Gynecol.

[5] Suehiro S, Yamashita S, Kamei M, Kawahara K, Daa T, Kashima K (2012). A case of breast metastasis from ovarian cancer diagnosed as primary breast cancer preoperatively. J Jpn Surg Assoc.

[6] Takao W, Sakurai M, Shikama A, Tasaka N, Nakao S, Ochi H (2016). A case of ovarian cancer serous carcinoma stage 4B with breast metastasis from the first treatment. Jpn J Gynecol Oncol.

[7] Ikari A, Tanaka S, Nitta T, Tanishima H, Horiuchi T, Hoshida Y (2017). A case of breast metastasis from ovarian cancer with calcification. J Jpn Surg Assoc.

[8] Kimura F, Oda G, Hanaoka M, Nakagawa T, Bee Y, Kubota K (2018). Ovarian cancer breast metastasis, A case report of intramammary lymph node metastasis from ovarian cancer experienced in our hospital and review of the literature. Jpn J Breast Cancer.

[9] Bloss JD, Liao SY, Buller RE, Manetta A, Berman ML, McMeekin S (1993). Extraovarian peritoneal serous papillary carcinoma: a case–control retrospective comparison to papillary adenocarcinoma of the ovary. Gynecol Oncol.

[10] Report by the Japan Obstetrics and Gynecology Society Gynecology Oncology Committee (2012). annual report of patients. Acta Obstet Gynaecol Jpn.

[11] Alvarado-Cabrero I, Young RH, Vamvakas EC, Scully RE (1999). Carcinoma of the fallopian tube: a clinicopathological study of 105 cases with observations on staging and prognostic factors. Gynecol Oncol.

[12] Komiyama S, Katabuchi H, Mikami M, Nagase S, Okamoto A, Ito K (2015). Japan Society of Gynecologic Oncology Guidelines 2015 for the treatment of ovarian cancer including primary peritoneal cancer and fallopian tube cancer.

[13] National Comprehensive Cancer Network: NCCN Clinical Practice Guidelines in oncology. Ovarian cancer including fallopian tube cancer and primary peritoneal cancer, version 1. 2020. https://www.nccn.org/professionals/physician_gls/pdf/ovarian.pdf. Accessed 6 Apr 2020.

[14] Wright AA, Bohlke K, Armstrong DK, Bookman MA, Cliby WA, Coleman RL (2016). Neoadjuvant chemotherapy for newly diagnosed, advanced ovarian cancer: society of gynecologic oncology and American Society of Clinical Oncology Clinical Practice Guideline. J Clin Oncol.

[15] Burger RA, Sill MW, Monk BJ, Greer BE, Sorosky JI (2007). Phase II trial of bevacizumab in persistent or recurrent epithelial ovarian cancer or primary peritoneal cancer: a Gynecologic Oncology Group Study. J Clin Oncol.

[16] Aletti GD, Powless C, Bakkum-Gamez J, Wilson TO, Podratz KC, Cliby WA (2009). Pattern of retroperitoneal dissemination of primary peritoneum cancer: basis for rational use of lymphadenectomy. Gynecol Oncol.

[17] Menczer J, Chetrit A, Barda G, Lubin F, Fishman A, Dgani R (2006). Primary peritoneal carcinoma—uterine involvement and hysterectomy. Gynecol Oncol.

[18] Tarin D, Prie JE, Kettlewell MG, Souter RG, Vass AC, Crossley B (1984). Mechanisms of human tumor metastasis studied in patients with peritoneovenous shunts. Cancer Res.

[19] Hajdu SI, Urban JA (1972). Cancers metastatic to the breast. Cancer.

[20] Nakamura H, Takehara K, Kusumoto S, Sasaki A, Yamazaki T, Samura O (2012). A case of cervical serous adenocarcinoma. Hiroshima Bull Jap Soc Clin Cytol.

[21] Sekine M, Nagata H, Tsuji S, Hirai Y, Fujimoto S, Hatae M (2001). Mutational analysis of *BRCA1* and *BRCA2* and clinicopathologic analysis of ovarian cancer in 82 ovarian cancer families: two common founder mutations of *BRCA1* in Japanese population. Clin Cancer Res.

[22] National Comprehensive Cancer Network: NCCN Clinical Practice Guidelines in Oncology Genetic/Familial High-Risk Assessment. Breast, ovarian, and pancreatic, version 1. 2020. https://www.nccn.org/professionals/physician_gls/pdf/genetics_bop.pdf. Accessed 16 Aug 2020. 10.6004/jnccn.2021.0001 33406487

[23] Balmana J, Diez O, Rubio IT, Cardoso F (2011). *BRCA* in breast cancer: ESMO Clinical Practice Guidelines. Ann Oncol.

[24] Mirza MR, Monk BJ, Herrstedt J, Oza AM, Mahner S, Redondo A (2016). Niraparib maintenance therapy in platinum-sensitive, recurrent ovarian cancer. N Engl J Med.

[25] Coleman RL, Oza AM, Lorusso D, Aghajanian C, Oaknin A, Dean A (2017). Rucaparib maintenance treatment for recurrent ovarian carcinoma after response to platinum therapy (ARIEL3): a randomised, double-blind, placebo-controlled, phase 3 trial. Lancet.

[26] Katsumata N, Yasuda M, Isonishi S, Takahashi F, Michimae H, Kimura E, Japanese Gynecologic Oncology Group (2013). Long-term results of dose-dense paclitaxel and carboplatin versus conventional paclitaxel and carboplatin for treatment of advanced epithelial ovarian, fallopian tube, or primary peritoneal cancer (JGOG 3016): a randomised, controlled, open-label trial. Lancet Oncol.

[27] Clamp AR, James EC, McNeish IA, Dean A, Kim JW, O’Donnell DM (2019). Weekly dose-dense chemotherapy in first-line epithelial ovarian, fallopian tube, or primary peritoneal carcinoma treatment (ICON8): primary progression free survival analysis results from a GCIG phrase 3 randomised controlled trial. Lancet.

